# Auxo-Endocrinological Approach to Celiac Children

**DOI:** 10.3390/diseases3020111

**Published:** 2015-06-19

**Authors:** Mauro Bozzola, Cristina Meazza, Alberto Villani

**Affiliations:** 1 Internal Medicine and Therapeutics Department, Pediatrics and Adolescentology Unit, University of Pavia, Fondazione IRCCS San Matteo, Viale Golgi 19, 27100 Pavia, Italy; 2 Internal Medicine and Therapeutics Department, Pediatrics and Adolescentology Unit, University of Pavia, Fondazione IRCCS San Matteo, Viale Golgi 19, 27100 Pavia, Italy; E-Mail: c.meazza@smatteo.pv.it; 3 Department of Pediatrics, Pediatric and Infectious Diseases Unit, Bambino Gesù Children Hospital, IRCCS, Piazza Sant’Onofrio 4, 00165 Rome, Italy; E-Mail: alberto.villani@opbg.net

**Keywords:** celiac disease, children, endocrine diseases, linear growth

## Abstract

Celiac disease is a permanent genetically determined intolerance to gluten that generally presents with gastrointestinal symptoms in young children and extraintestinal manifestations (endocrinological, dermatological, neurological, *etc*.) later. Furthermore, many studies demonstrate the close association between celiac and endocrine diseases, including growth and pubertal disorders, type I diabetes mellitus and autoimmune thyroid diseases, probably due to the presence of a common genetic predisposition. Follow-up for celiac children after the start of gluten-free diet is mandatory to avoid complications such as growth hormone deficiency. The present review deals with the problem of the diagnosis of endocrine-associated diseases in celiac children and gives suggestions for correct management and follow-up of these patients.

## 1. Introduction

Celiac disease (CD) is a systemic immune-mediated disorder triggered by dietary gluten in genetically susceptible subjects [1]. It is the most clinically relevant condition in which genetic predisposition and environmental factors interact to trigger an autoimmune response, and is the only human autoimmune condition in which the causative antigen is known.

Treatment of CD consists of a gluten-free diet (GFD) which determines remission of intestinal damage, and protects against the risk of developing autoimmune diseases associated with CD and “normalizes” the mortality risk related to the disease itself [2,3].

Classically, children with CD present with typical malabsorption signs and symptoms such as failure to thrive, diarrhea, abdominal distension, muscle wasting, poor appetite and irritability. Recent evidence points to the occurrence of extraintestinal manifestations (Table 1), such as iron and folic acid deficiency with or without anemia, dermatitis herpetiformis, delayed puberty, short stature, enamel defects, and recurrent aphthous stomatitis, before the diagnosis of celiac disease or during its management.

**Table 1:** Extraintestinal manifestations of CD.

**SKELETAL SYSTEM:**
Osteopenia/osteoporosis Arthritis Enamel defects
**ENDOCRINE SYSTEM:**
Short stature Delayed puberty Infertility
**CENTRAL NERVOUS SYSTEM:**
Epilepsy with occipital calcifications
Epilepsy with occipital calcifications Depression Ataxia
**OTHER SYSTEMS:**
Myelopathy Iron deficiency anemia refractory to oral iron supplementation Hypertransaminasemia Dermatitis herpetiformis

Pediatricians frequently encounter the problem of short stature or stunted growth rate in CD subjects. In fact, the prevalence of CD in patients evaluated for short stature varies between 2% and 10% [4,5,6,7].

Indeed, during the evaluation of a short child the first step is the exclusion of CD, which may be responsible for growth failure [8]. After the start of a GFD, catch-up growth is generally observed, and the celiac child usually returns to his/her normal growth curve for weight and height within 1–2 years. However, an endocrinological investigation including an evaluation of GH secretion should be performed in CD children who show no catch-up growth after at least one year of a strict GFD, and after seronegativity for anti-tissue transglutaminase and/or anti-endomysial antibodies have been confirmed. In subjects with CD and growth hormone deficiency (GHD), substitutive therapy with GH might be promptly started at standard doses, in order to obtain complete catch-up growth. The long-term effects of GH therapy in children following a strict GFD are similar to those observed in children with idiopathic GHD [9].

Finally, the existence of a close relationship between CD and autoimmune diseases, such as thyroid disorders and diabetes mellitus type I, is suggested by the fact that CD is an autoimmune disorder. The pathogenetic mechanism is still not completely known and only partly linked to the increase of intestinal permeability.

The present review contains some suggestions for the correct diagnostic work-up for CD in subjects who, even in the absence of gastrointestinal symptoms, show extraintestinal symptoms such as short stature and/or delayed puberty. The management of short stature and other endocrine related conditions in celiac children and adolescents is discussed.

## 2. General Auxological Approach

**Figure 1:**
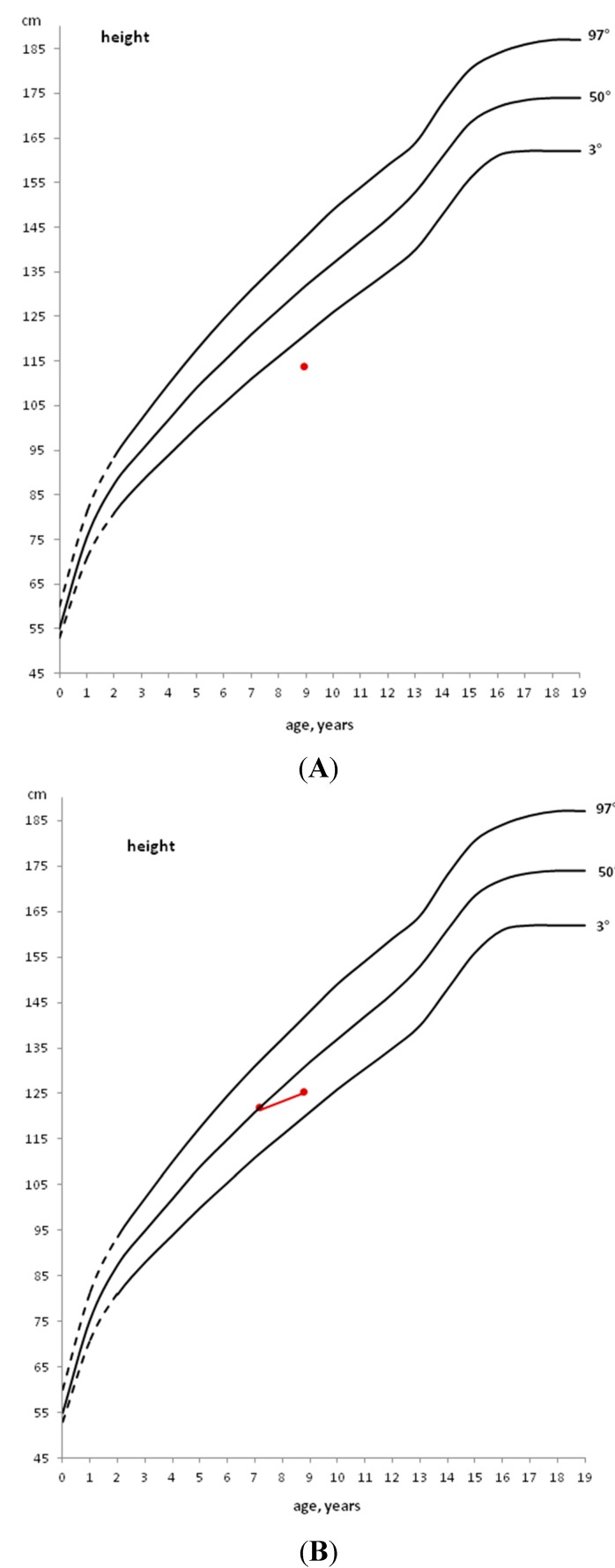

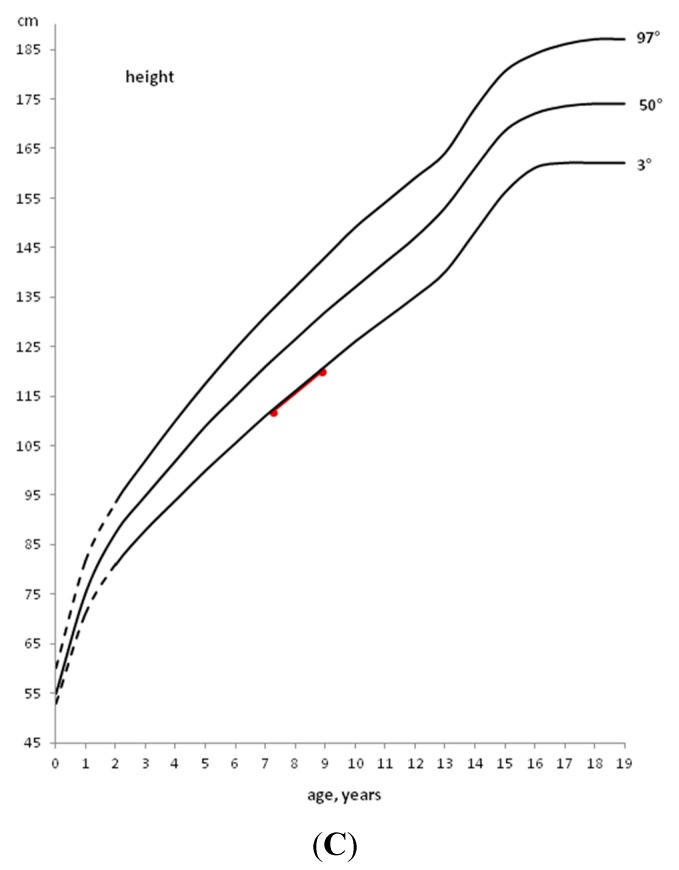
Example of patients with height below the 3rd percentile (**A**), with height, initially above the 50th percentile, that decreases to a lower percentile (**B**) and with height within the 3rd percentile that does not decrease to a lower percentile (**C**).

In a child with a stature below the 3rd percentile (Figure 1, Panel A) or with growth deceleration (Figure 1, Panel B), a diagnostic work-up should be started to identify any pathological cause [10].

If a pre-pubertal child shows a stature close to the 3rd percentile, but grows along the same percentile, only an annual growth follow-up is indicated (Figure 1, Panel C).

If, instead, a child grows on a percentile lower than his/her genetic target or when the first pubertal signs (appearance of the breast development in the female and increased testicular volume in the male) are already evident, the diagnostic work-up should be started immediately, without waiting for a growth rate decrease [10].

First of all, it is necessary to evaluate whether the short stature is clinically suggestive of a normal variant of the growth pattern (e.g., familial short stature or constitutional delay of growth and puberty) or of a specific pathology (e.g., achondroplasia, hormonal dysfunction). In the differential diagnosis, to discern between familial short stature and/or constitutional delay of growth and puberty and endocrine conditions, a bone age evaluation is useful, since it indicates the subject’s growth potential.

Therefore, the next step is the exclusion of other possible conditions responsible for growth failure including kidney or liver disease, skeletal disorders, subclinical hypothyroidism, intestinal malabsorption such as Crohn’s disease and CD. It is accepted practice to exclude celiac disease before evaluating GH secretion as well, in a short child in whom GHD is suspected. In fact, pathological GH responses to pharmacological tests have not been confirmed following the institution of the GFD [11,12]. Insulin-like growth factor I (IGF-I), which is considered the peripheral GH mediator, is low in patients with insufficient GH secretion, but is not a discriminating factor in the evaluation of GH secretion, since its level is influenced also by the nutritional status of the subject.

Furthermore, in a patient with delayed appearance of pubertal signs (such as absence of the mammary gland over 13 years of age in female subjects and >4 mL testicular volume in male subjects over 15 years of age), it is necessary to exclude CD. In fact, after resolution of a pathological condition which may have determined growth deceleration, spontaneous catch-up growth is generally observed.

**Figure 2:**
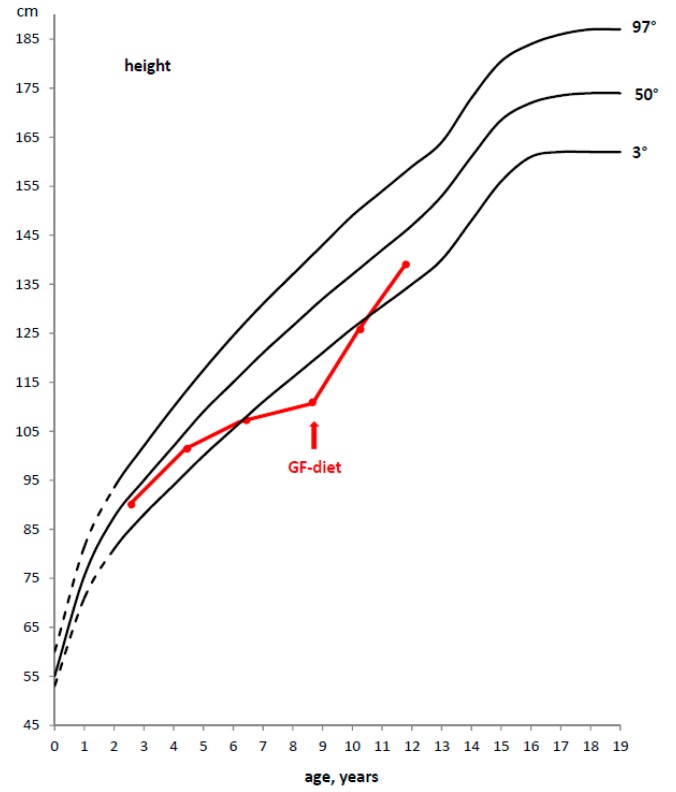
Example of a deceleration of growth rate in a CD child and catch-up growth after the introduction of a GFD.

Also in CD, after the start of a GFD, a significant catch-up growth is generally observed. Therefore, the celiac child usually returns to his/her normal growth curve for weight and height within 1–2 years (Figure 2).

Therefore, a careful auxological follow-up is necessary to verify growth and weight catch-up, in addition to the annual evaluation of serological negativity. In fact, to confirm dietary compliance the annual monitoring of anti-tTG-IgA is recommended, in consideration of its high sensitivity and specificity. If after 1–2 years of a GFD the subject does not show clear catch-up growth, in the presence of seronegativity for specific celiac antibodies, the evaluation of GH secretion in response to at least two pharmacological stimuli is mandatory. In fact, it has been observed that 0.23% of children with short stature show an association between CD and GHD [13].

In the presence of a normal GH response to at least one pharmacological stimulus and IGF-I values within the normal range for sex and age, the auxological follow-up should be repeated every year. In the case of a pathological response (*i.e.*, GH peak < 8 ng/mL to both stimulus tests) and in the presence of a negative specific serology, once the basal levels of FT4, TSH, ACTH and cortisol have been verified, substitutive therapy with recombinant human GH should be started as in patients with idiopathic GHD [14]. Nuclear magnetic resonance imaging of the brain is required to exclude any morphological abnormality of the hypothalamic-pituitary region. In the rare cases of GHD associated with a deficiency in one or more pituitary hormones (TSH, ACTH, LH, FSH, ADH), correct hormonal secretion should be restored by substitutive therapy with the missing hormones before starting GH treatment. A deficit in pituitary gonadotropins, LH and FSH, may be assessed only during the pubertal period when in normal children a pubertal gonadotropins increase.

**Figure 3:**
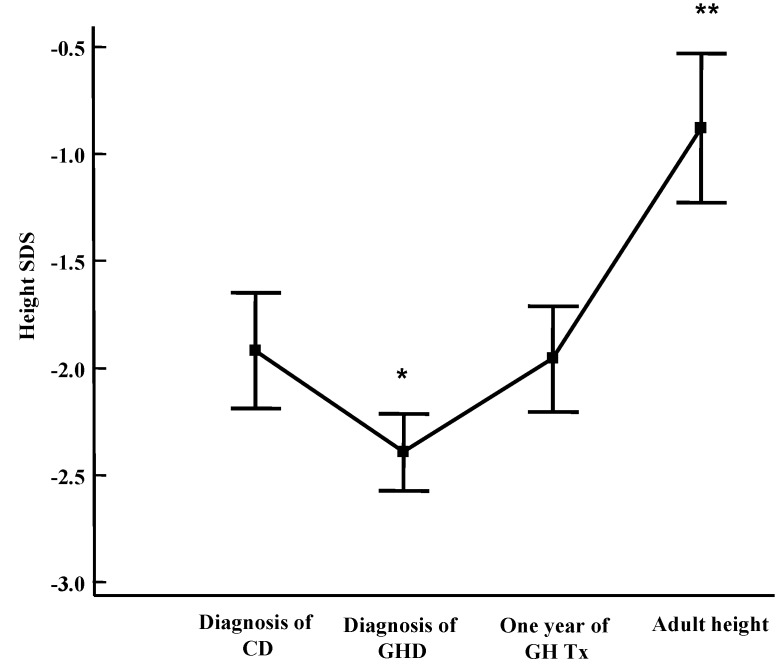
Height Standard Deviation Score (SDS) in CD-GHD patients at CD diagnosis, at GHD diagnosis, after 1 year of GH replacement therapy and at final height. Results are expressed as mean ± SEM (Standard Error of Mean) [9].

Celiac patients with GHD should be treated with the same GH dosage as patients with idiopathic GHD (0.23–0.25 mg/kg/week s.c.), administered in the evening before sleeping to mimic the physiological night-time elevation of the hormone. In the case of associated hormonal deficiencies, the doses of levothyroxine, hydrocortisone, estradiol or testosterone enantate according to gender and desmopressin are the same ones used in GHD patients. In celiac patients with GHD, the response to substitutive treatment is similar to that of subjects with idiopathic GHD [9,12,13]. Both height and growth velocity significantly improved during the therapy, confirming that the absence of catch-up growth after a GFD was not due to malnutrition, but to low GH secretion. The growth rate increases, especially during the first year of GH therapy, and then decreases, although it always remains above pre-treatment values [13]. Moreover, we have previously shown that CD children treated for associated GHD reach normal final height [9] (Figure 3).

Finally, compliance to GFD is very important in order to obtain a good response to GH therapy. Clinical results suggest, in fact, that CD patients with GHD do not respond to hormonal substitutive therapy if they do not observe a strict GFD [9].

## 3. Development of Endocrine Autoimmune Conditions in Children with CD

The existence of a close association between CD and endocrine autoimmune diseases such as autoimmune thyroiditis, type 1 diabetes mellitus (T1DM) and pubertal disorders has been widely demonstrated. A common genetic predisposition, such as HLA-DQ2 or DQ8 haplotypes, seems to partially explain the association between CD and autoimmune diseases. The development of multiple autoimmunity may be due to shared epitopes between an environmental agent and common antigens present in several endocrine tissues. In CD, the ingestion of gliadin causes an immune response against the enzyme tissue transglutaminase, which could share epitopes with other molecules present especially in endocrine glands [15,16].

Furthermore, in CD patients the increased risk of developing other autoimmune diseases seems to be related to the duration of gluten exposure [17].

### 3.1. Thyroiditis

The thyroid is frequently affected in CD patients, with highly variable percentages of hypo- and hyperthyroidism [18]. The frequent association of CD with autoimmune thyroid disorders is based on shared immunopathological mechanisms linked to the haplotype HLA-B8 and -DR3, which are more frequent in these patients compared with the general population. The prevalence of association between thyroiditis and CD varies from 4% to 14% and the most frequent association is between CD and Hashimoto thyroiditis [19]. Furthermore, early markers of thyroid autoimmune involvement, such as anti-thyroid peroxidase antibodies (anti-TPO) or thyroid echographic alterations with euthyroidism, have more frequently been observed in CD patients compared with the healthy population [20]. On the contrary, some authors reported that the presence of antithyroid antibodies in children with celiac disease has a low predictive value for the development of thyroid hypofunction during a three year-surveillance period [21].

However, relying on our personal experience, in CD subjects, we suggest periodically monitoring thyroid function (FT4 and TSH) and autoantibodies (anti-thyroid thyroglobulin antibodies (anti-TG) and anti-TPO, in the case of a TSH increase.

In the presence of anti-thyroid antibodies, if Hashimoto thyroiditis is suspected, a thyroid echography should be performed to evaluate the structure of the thyroid parenchyma. In the rare case of reduced FT4 and increased TSH, low-dosage treatment with levothyroxine (12.5–25 µg per day, to be taken orally at least 30 min before breakfast) should be started, and increased to the full dosage in 1–2 months, with biannual monitoring of FT4 and TSH, without considering anti-TPO and anti-TG levels. On the other hand, in some patients with autoimmune thyroiditis and CD following a GFD, the dose of replacement therapy can be progressively reduced and, in a few cases, the diet could normalize a subclinical hypothyroidism [19].

### 3.2. Diabetes

Another widely documented association is that between CD and T1DM. In fact, CD is observed at a higher frequency in T1DM patients with prevalence rates up to 16.4% compared to 1% prevalence in the general population [22]. Genetic studies report a higher frequency of HLA-B8, -DR3 and -DQW2 both in patients with T1DM and in those with CD in comparison with the general population. Furthermore, alterations in β cells and enterocytes seem to be due to the same factors, such as proinflammatory cytokines (e.g., interferon-γ and TNF-α). In most cases CD is diagnosed months or years after the onset of T1DM, although at the onset of T1DM a temporary rise in antigliadin/antitransglutaminase antibodies is described in 3%–4% patients. The probability of developing CD increases with the duration of diabetes [23]. Predictive parameters of the risk of developing T1DM include the presence of anti-insulin antibodies (IA2), anti-glutammic acid decarboxylase antibodies (GAD), anti-zinc transporter 8 antibodies (ZnT8). At the onset of T1DM, and annually thereafter, it is common practice to evaluate CD serology and, in the case of abnormal anti-tTG levels, a duodenal-jejunal biopsy is required to detect any intestinal damage suggestive of CD [24]. In cases of potential CD, when a positive serological test does not correspond with any histological intestinal mucosal alterations (Marsh stage 0 or 1), an annual auxo-endocrinological and serological follow-up are recommended.

### 3.3. Other Autoimmune Diseases

In some CD patients, hypogonadism has been described with a delay in the onset of puberty and retarded menarche in untreated CD girls. Similarly, boys show tissue resistance to androgens characterized by reduced serum levels of dihydrotestosterone and increased levels of luteinizing hormone. Probably, a selective malabsorption of micronutrients (zinc, iron, folic acid, vitamins) important for the metabolism, for the functionality of sex hormone receptors and for autoimmune processes may have a role in the pathogenetic mechanisms of hypogonadism [25].

In CD patients, an autoimmune hypophysitis resulting in hypopituitarism and impaired growth could be found. In fact, an autoimmune syndrome may hamper the growth response to a GFD. Some authors have detected anti-pituitary and anti-hypothalamus autoantibodies in CD and GHD children without catch-up growth after GFD, suggesting the onset of autoimmune hypophysitis involving somatotropic cells [26,27]. Therefore, the paediatric endocrinologist could also consider testing for anti-pituitary and anti-hypothalamus antibodies in all patients with CD-associated GHD.

In conclusion, taking into account the high incidence of endocrine disorders in CD, and that an early diagnosis and treatment of CD might protect against the development of autoimmune conditions, annual serological screening for CD is recommended in all patients with T1DM or other autoimmune endocrine diseases [2].

## 4. Conclusions

Children with CD should be monitored regularly to verify a normal growth and pubertal development, appearance of symptoms, and adherence to GFD. Evaluating adherence to GFD should be based on a combination of personal and dietary history and serology.

GH secretion should be evaluated in CD children who show no catch-up growth after at least one year of a strict GFD, and after seronegativity for anti-tissue transglutaminase and/or anti-endomysial antibodies have been confirmed. In subjects with CD and GHD, substitutive therapy with GH should be administered at standard doses and should be started promptly, in order to achieve complete catch-up growth. The long-term effects of GH therapy in children who follow a strict GFD are similar to those observed in children with idiopathic GHD.

Furthermore, CD patients should be monitored annually also for the occurrence of other autoimmune endocrine diseases, in particular thyroiditis.
